# Extensive targeted metabolomics analysis reveals the identification of major metabolites, antioxidants, and disease-resistant active pharmaceutical components in *Camellia tuberculata* (*Camellia* L.) seeds

**DOI:** 10.1038/s41598-024-58725-0

**Published:** 2024-04-15

**Authors:** Zhaohui Ran, Zhi Li, Xu Xiao, Chao Yan, Mingtai An, Juyan Chen, Ming Tang

**Affiliations:** 1 College of Forestry, Guizhou University, Guiyang, China; 2 College of Forestry, Jiangxi Agricultural University, Nanchang, China; 3 Guizhou Academy of Forestry Sciences, Guiyang, China; 4 Biodiversity and Nature Conservation Research Center, Guizhou University, Guiyang, China

**Keywords:** *Camellia tuberculata*, UPLC/ESI-Q TRAP-MS/MS, Seeds, Metabolites, Antioxidant activity, Disease resistance, Molecular biology, Plant sciences

## Abstract

Sect. *tuberculata* plant belongs to the *Camellia* genus and is named for the “tuberculiform protuberance on the surface of the ovary and fruit”. It is a species of great ornamental value and potential medicinal value. However, little has been reported on the metabolites of *C. tuberculata* seeds. Therefore, this study was conducted to investigate the metabolites of *C. tuberculata* seeds based on UPLC/ESI-Q TRAP-MS/MS with extensively targeted metabolomics. A total of 1611 metabolites were identified, including 107 alkaloids, 276 amino acids and derivatives, 283 flavonoids, 86 lignans and coumarins, 181 lipids, 68 nucleotides and derivatives, 101 organic acids, 190 phenolic acids, 10 quinones, 4 steroids, 17 tannins, 111 terpenoids, and 177 other metabolites. We compared the different metabolites in seeds between HKH, ZM, ZY, and LY. The 1311 identified different metabolites were classified into three categories. Sixty-three overlapping significant different metabolites were found, of which lignans and coumarins accounted for the largest proportion. The differentially accumulated metabolites were enriched in different metabolic pathways between HKH vs. LY, HKH vs. ZM, HKH vs. ZY, LY vs. ZY, ZM vs. LY and ZM vs. ZY, with the most abundant metabolic pathways being 4, 2, 4, 7, 7 and 5, respectively (p < 0.05). Moreover, among the top 20 metabolites in each subgroup comparison in terms of difference multiplicity 7, 8 and 13. ZM and ZY had the highest phenolic acid content. Ninety-six disease-resistant metabolites and 48 major traditional Chinese medicine agents were identified based on seven diseases. The results of this study will not only lead to a more comprehensive and in-depth understanding of the metabolic properties of *C. tuberculata* seeds, but also provide a scientific basis for the excavation and further development of its medicinal value.

## Introduction

Plant metabolites are chemical substances produced by plants during growth and development. They play an important role in regulating metabolism and adaptation to the environment in plants^1,2^. These compounds include phytochromes, growth hormones, phenolic acids, basic amino acids, monoterpenoids, etc. With the development of modern analytical techniques, more and more plant metabolites are being identified and characterized. However, although thousands of plant metabolites have already been identified, there are still many unknown metabolites waiting to be discovered^3,4^. The roles of metabolites are also complex and diverse and may include plant protection, lubrication, resistance to pathogens, combating interspecies competition, and forming ecosystem interactions. For example, tea polyphenols can improve the antibacterial capacity of the immune system and regulate liver and heart function^5^; isoflavones in soy may help reduce breast cancer risk in women^6^; and some plants with defensive metabolites can repel herbivores and parasites and attract beneficial insects^7^. For edible plants, differences in metabolites can also affect their taste, color, nutrient content, and health effects^8–11^. Therefore, understanding the differences in metabolites and their roles in different plants or different species of the same plant can not only enhance our understanding of ecosystems, plant physiology, and chemistry, but also have important implications for human health protection and food industry development.

*Camellia* L. is an important flowering plant with great biodiversity and economic value. In recent years, researchers have used metabolomics techniques to analyze and characterize in detail the major metabolites of the genus *Camellia*. For example, metabolomics analysis of *Camellia japonica* has been^12^ revealed that its flowers contain high amounts of kaempferol, which have a strong antioxidant and anti-inflammatory effect and should be an important source for the production of natural health products and medicines. Metabolomics of *Camellia oleifera*^13^ showed that it is rich in unsaturated fatty acids such as oleic acid, linoleic acid, and linolenic acid, which have important health effects on human health and whose applications are gaining increasing attention. Metabolomic analyzes have been conducted for various tea varieties such as green tea, black tea, oolong tea, and black tea^14–17^, which not only revealed the major compounds in tea but also provided new insights into the health effects of tea, thus providing new ideas for tea product development and health care. In addition, metabolomic studies can also be used to reveal changes in the metabolic pathways of the genus *Camellia*. For example, the metabolomics approach can be used to study in depth the secondary metabolites in the genus *Camellia*, and some of them have been found to have biological activities such as antioxidant, anti-inflammatory, and anticancer properties^18^. Meanwhile, metabolomics can also be used to reveal the response mechanisms of the *Camellia* genus to adversity. For example, metabolomics studies under drought stress conditions have revealed some response pathways to adversity and changes in response pathways in *Camellia*, and promoted a deep understanding of the adaptability of the *Camellia* genus^19^. In summary, with the development and application of metabolomics technology, the metabolomics study of the genus *Camellia* has been intensified, providing richer data and methods for the study of taxonomy, physiological and biochemical mechanisms, and nutrient composition of the genus *Camellia*.

*Camellia tuberculata* Chien belongs to *Camellia* and is named for the "tuberculiform protuberance on the surface of the ovary and fruit"; therefore, it is considered a specialized taxon within sect. *Tuberculata* Chang Tax that has retained the primitive form^20^. It is divided into 17 species according to the classification of Zhang Hongda et al. It is found only in the southwestern part of China and is an endemic taxon in the subtropical region of China^21,22^. Current research on the genus *Camellia* focuses mainly on economic plants (Sect. *Thea* Dyer in Hook.), seed oil plants (Sect. *Oleifera* Chang Tax. and Sect. *Paracamellia* Sealy Rev.), horticultural green plants (Sect. *Chrysantha* Chang), and medicinal plants for drug production (Sect. *Thea* and Sect. *Oleifera*). To date, relatively few studies have been conducted on plants of sect. *Tuberculata*. With only a few studies on taxonomic revision, chloroplast genome characteristics, seed germination, and chemical composition^20–27^, the major metabolites and active compounds are not yet clear, which may hinder further development and utilization of sect. *Tuberculata* plants is hindered. Therefore, it is necessary to conduct a metabolomic analysis.

In this study, the metabolites of the seeds of four *C. tuberculata* cultivars were identified and quantified using a metabolomics approach based on ultra-performance liquid chromatography-electrospray ionization-triple quadrupole tandem mass spectrometry (UPLC/ESI-Q TRAP-MS/MS). The bioactive potential of the identified metabolites was further evaluated by performing DPPH, CUPRAC, FRAR, total flavonoid and total phenolic acid tests. In addition, the identified metabolites were analyzed for seven common key components of disease resistance based on the Traditional Chinese Medicine Systematic Pharmacology Database and Analysis Platform (TCMSP), and the results of these analyzes will support basic research on their medicinal value. In summary, metabolomic, antioxidant, and network pharmacological analyzes were performed on *C. tuberculata* seeds in this study to provide a reference point and guidance for in-depth investigation and to support the development of the medicinal and health value of the plant with sound data.

## Materials and methods

### Metabolites detection in the seeds of four *C. tuberculata*

**Figure 1:**
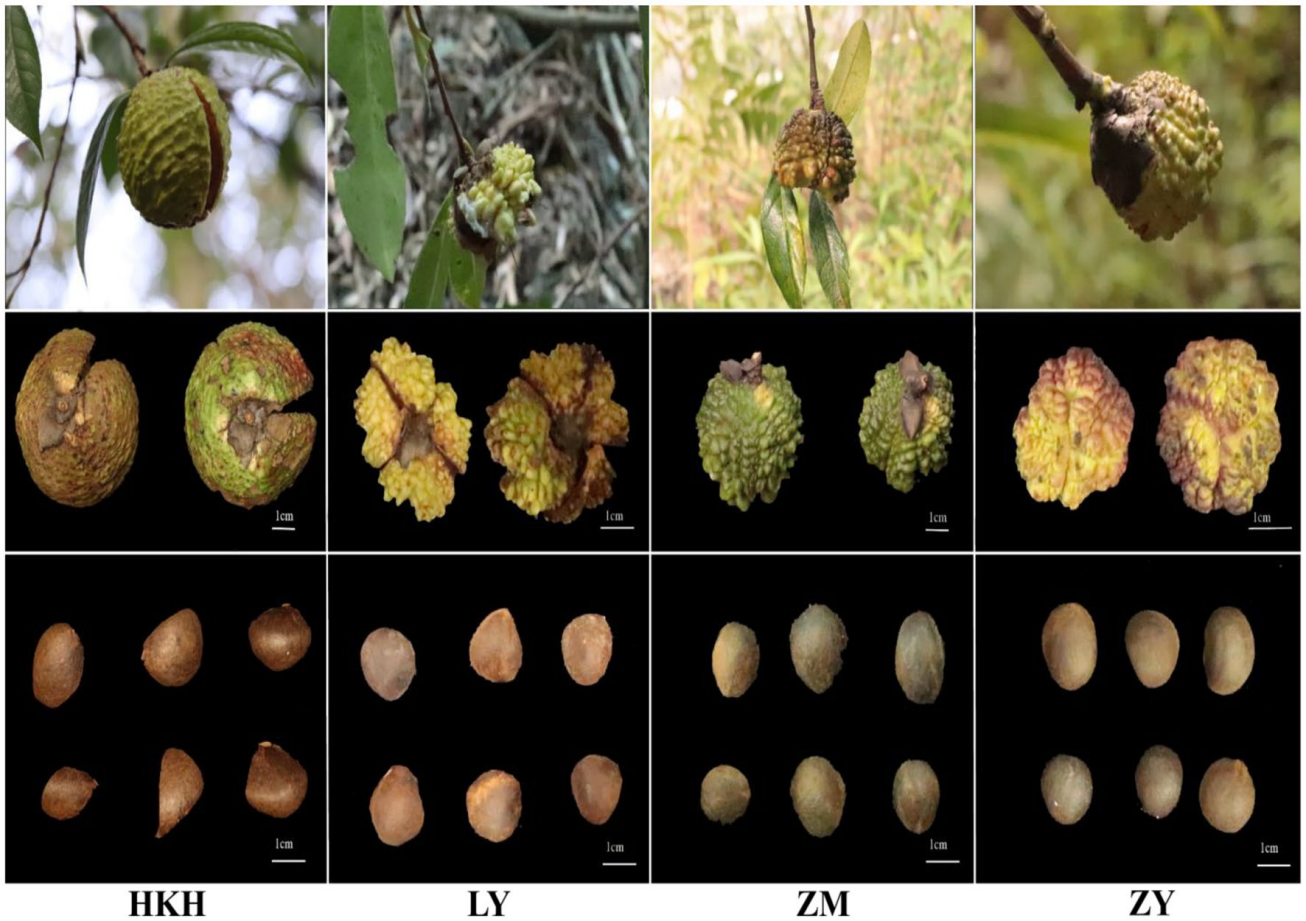
Seeds of four *C. tuberculata* varieties. (HKH, *Camellia rubituberculata*; LY, *Camellia leyeensis*; ZM, *Camellia atuberculata*; ZY, *Camellia rhytidocarpa*).

Four *C. tuberculata* species (HKH, LY, ZM, and ZY) were used in this study and differed in seed shape, seed size, and original collection site (Fig. 1). All four *C. tuberculata* plants were collected in the field, including *C. rubituberculata* Chang et Yu from Shi Goose Factory, Xingren City, Guizhou Province (105° 21′ 08.10″ E, 25° 43′ 50.08″ N), *C. leyeensis* Chang et Y. C. Zhong from Yachang, Leye County, Guangxi Province (106° 17′ 31.48″ E, 24° 51′ 29.98″ N), *C. atuberculata* Chang from Yuan Hou DaWuji, Chishui, Guizhou Province (105° 55′ 59.51″ E, 28° 20′ 27.88″ N), and *C. rhytidophylla* Y. K. Li et M. Z. Yang were collected from Baixiang Forest, Shuangshan Village, Feng San Town, Kaiyang County, Guizhou (107° 4′ 26.55″ E, 27° 6′ 34.84″ N). Three plants of each cultivar were randomly collected from three biological replicates. The collected seeds were packed in polyethylene bags and stored in a refrigerator at – 20 °C until the extraction of metabolites.

### Sample preparation and extraction

Biological samples were placed in a lyophilizer (Scientz-100F) for rapid freeze drying, and then the samples were ground (30 Hz, 1.5 min) to a powder using a grinder (MM 400, Retsch). Then weigh 50 mg of the sample powder using an electronic balance (MS105DΜ) and add 1200 μL of 70% methanolic aqueous internal standard extract precooled at – 20 °C (Samples weighing less than 50 mg were added to the extract at a ratio of 1200 μm of extract per 50 mg of sample). Shake once every 30 min for 30 s for a total of 6 times. After centrifugation (rotation speed 12,000 rpm, 3 min), the supernatant was aspirated and the sample was filtered through a microporous membrane (0.22 μm pore size) and stored in the injection vial for UPLC-MS/MS analysis^28^.

### UPLC conditions and ESI-Q TRAP-MS/MS

#### UPLC conditions

Sample extracts were analyzed using a UPLC-ESI-MS /MS system (UPLC, ExionLC™AD' https://sciex.com.cn/; MS, Applied Biosystems 4500 Q TRAP, https://sciex.com.cn/)^29^. The UPLC conditions are described as follows: (1) Column: Agilent SB-C18 reversed-phase column with a length of 100 mm, an inner diameter of 2.1 mm, and a particle size of 1.8 µm was used, (2) Mobile phase A: methanol–water (90:10, containing 0.1% formic acid and 0.1% acetic acid), pH adjusted to 3.5, (3) Mobile phase B: pure methanol, (4) Gradient elution conditions: start with 10% mobile phase B; 0–0.5 min, linear gradient from 10 to 40% mobile phase B; 0.5–3 min, mobile phase B maintained at 40%; 3–3.5 min, linear gradient from 40 to 90% mobile phase B; 3.5–4 min, mobile phase B maintained at 90%; 4–4.5 min, linear gradient from 90% back to 10% mobile phase B; 4.5–6 min, mobile phase B maintained at 10%, (5) Flow rate: 0.3 mL/min, (6) Injection volume: 3 µL, and (7) Temperature: room temperature. The efflux was alternatively applied to an ESI-triple quadrupole-linear ion trap (QTRAP)- MS.

#### ESI-Q TRAP-MS/MS

The ESI source parameters were first set: ion spray voltagae (IS) 5500 V(positive ion mode)/-4500 V(negative ion mode); other set parameters were source temperature 550 °C, drying gas flow rate 10 L/min, evaporation secondary gas flow rate 20 L/min, and collision energy 35 eV. Then the ion reaction monitoring (MRM) mode was selected using mass spectrometry scanning, and the target metabolite, the specific mass-to-charge ratio (*m/z*) of the target metabolite, and the internal standard ion were then set using QQQ scans in MRM mode. The precursor ion (Q1) and product ion (Q3) are selected for ion section transfer by a Based Peak Intensity (BPI) scan. Operational parameters such as ion source gas, scan time, and collision energy are set to obtain optimal signal intensity and peak separation.

### Qualitative and quantitative metabolite analyses

Based on a self-built database MWDB V2.0 (Metware database), substance qualitative analysis is performed on the basis of secondary spectral information, and the analysis removes isotopic signals, repetitive signals containing K^+^ ions, Na^+^ ions, and NH4^+^ ions, and repetitive signals of fragment ions that are themselves other substances of greater molecular weight.

Metabolite quantification is accomplished using triple quadrupole mass spectrometry in the multiple reaction monitoring (MRM) mode of analysis, in which the quadrupole first screens the precursor ions (parent ions) of the target substance, excluding the ions corresponding to other molecular weight substances to initially eliminate interference; the precursor ions are broken by collision chamber-induced ionization to form Many fragment ions are formed after the precursor ions are induced to ionize by the collision chamber, and the fragment ions are then filtered through a triple quadruple rod to select the desired one characteristic fragment ion, eliminating non-target ion interference and making the quantification more accurate and reproducible. After obtaining the metabolite spectral analysis data from different samples, the peak areas of all substance chromatographic peaks were integrated, and the integration of the mass spectra of the same metabolite in different samples among them was corrected. In order to compare the differences in substance content of each metabolite in different samples among all detected metabolites, based on the information of metabolite retention time and peak shape, we corrected the chromatographic peaks of each metabolite detected in different samples to ensure the accuracy of qualitative and quantitative analysis. The integral correction results of the quantitative analysis of randomly selected metabolites in different samples are shown in Figure S1.

### KEGG annotation and enrichment analysis

The metabolites interact with each other in the organism and form different metabolic pathways. Thus, the identified metabolites were annotated using the KEGG Compound Database (http://www.kegg.jp/kegg/compound/)^30^. The annotated metabolites were then mapped to the KEGG Pathway Database (http://www.kegg.jp/kegg/pathway.html). The pathways with significantly regulated metabolites were then entered into the MSEA (Metabolite Sets Enrichment Analysis) and their significance was determined by the p-values of the hypergeometric test.

### Determination of the total phenolic content and total flavonoids content of plants

The method used to measure total plant flavonoids and total phenols was spectrophotometric^31^. The plant samples were first collected and cleaned, and then ground into a fine powder. Then about 0.5 g of the finely powdered sample was added to about 50 mL of extractant (60% ethanol) and shaken back at room temperature for 2 h. Then the extract was filtered through gauze and collected into a clear extract. A certain amount of the extract was taken and properly diluted with a solvent (distilled water) to a suitable concentration range. Finally, a series of standard solutions of known concentrations are prepared (TPC: 10 mg/mL Rutin standard solution, TFC: 1 mg/mL Tannic acid standard solution), and the absorbance of the extracts and standard solutions was measured at specific wavelengths (TPC: 470 nm, TFC: 760 nm) using a spectrophotometer. and their absorbance is measured according to the same measurement conditions. Then the concentrations of total flavonoids and total phenols in the samples were calculated based on the linear relationship between absorbance and known concentrations in the standard curves. For more details, please refer to the instructions for the total flavonoids and total phenols kit provided by Yuanxin Bio.

### Antioxidant activity of plants

#### CUPric ion reducing antioxidant capacity (CUPRAC)

The CUPRAC assay is a commonly used method for the determination of antioxidant activity and is performed as follows^32^: Firstly, 1 mL CuCl_2_ solution, 1 mL Neocuproine solution, 1 mL NH_4_Ac buffer solution (pH = 7.0), Volumes of 0.7 mL (extract) antioxidant sample solution and 0.4 mL H_2_O were added to the initial mixture so as to make the final volume 4.1 mL. The mixed solution was selected into the spectrophotometer cuvette together with the Gallic acid standard solution of known concentration, mixed, and the initial absorbance (A_0_) was recorded at 600 nm. Then, the sample solution to be measured was added to the mixed solution while it stood at room temperature for a period of time, usually 30 min. Finally, the absorbance (A) was recorded again at 600 nm. The antioxidant capacity was calculated by comparing the absorbance difference between the sample solution and the standard solution (Gallic acid).

#### 1,1-diphenyl-2-picrylhydrazyl (DPPH)

Firstly, 0.1 mM of DPPH solution was mixed with the sample solution to be tested in a certain ratio. The mixed solution was selected into the spectrophotometer cuvette together with the Trolox standard solution of known concentration, mixed well, and the initial absorbance (A_0_) was recorded at 517 nm. Then, the sample solution to be measured was added to the mixed solution while it stood at room temperature for a period of time, usually 30 min. Finally, the absorbance (A) was recorded again at 517 nm. The antioxidant capacity was calculated by comparing the difference in absorbance between the sample solution and the standard solution (Trolox)^33^.

#### Ferric-reducing antioxidant power (FRAP)

The steps of plant FRAP measurement were as follows^34^: First, a certain proportion of 3 mM HCl solution, 20 mM FeCl_3_ solution, and 0.3 M HAc buffer solution (pH = 3.6) were mixed to obtain the FRAP reagent solution. Then the sample solution to be measured was properly treated to reach the appropriate concentration, and the initial absorbance (A_0_) was recorded. The FRAP reagent solution was mixed with the sample solution to be tested in a certain ratio, and the absorbance (A) was recorded at 590 nm. Finally, the antioxidant capacity was calculated by comparing the absorbance difference between the sample solution and the standard solution (FeSO_4_).

### Identification of the active pharmaceutical ingredients for four major diseases-resistance in *C. tuberculata* seeds

TCMSP is an online molecular database platform for herbal medicines that can perform activity prediction, mechanism of action analysis, and multi-target network analysis of herbal ingredients^35^. One of them, CancerHSP, contains 2439 anticancer herbs and 3575 anticancer ingredients, which greatly facilitates anticancer mechanism analysis and drug synthesis. On this basis, we compared the discovered metabolites with the TCMSP library of compounds associated with diseases such as cancer, diabetes, hypertension, cardiovascular disease, thrombosis, atherosclerosis, and osteoporosis, and investigated the antidisease constituents in the seeds of *C. tuberculata* of TCMSP. The metabolites screened for resistance to seven major diseases were analyzed for active components. Related disease numbers, related target numbers, oral bioavailability, and drug likenesses were entered in TCMSP, and the results were tabulated. This will be an important reference for further analysis of the medicinal value.

### Data processing and statistics

All data was counted in Excel 2010. Pearson correlations of TPC, TFC, and antioxidant capacity of *C. tuberculata* seeds were tested by SPSS 16.0, and correlation graphs were plotted in Origin 2022. The raw signal data were normalized using Analyst 1.6.3 software. HCA, PCA, and PCC were performed in R version 4.3.1. Abbreviations appearing in this article are in Table S7.

### Statement

With regard to experimental research and field studies on plants, including the collection of plant material, we comply with relevant institutional, national and international standards and legislation.

## Results

### Metabolites detection in the seeds of four *C. tuberculata*

First, the mass spectrometry data were processed to obtain the total ion flux map and MRM metabolite detection multipeak map (Supplementary Fig. S2) of the mixed QC samples. Based on the local database, the metabolites of the samples were analyzed qualitatively and quantitatively by mass spectrometry. Then, the reproducibility of metabolite extraction and detection was analyzed by overlap analysis of the total ion flux maps of mass spectrometry detection analysis of different QC samples. The results showed a high curve overlap for the total ion flux of metabolite detection (Supplementary Fig. S3A,B), indicating that the signal stability of mass spectrometry for the detection of the same sample at different time points was good, and the high stability of the instrument provided an important guarantee for the reproducibility and reliability of the data. Finally, correlation analysis was performed between three replicates of each sample, and the results showed good homogeneity among the samples (Supplementary Fig. S3C). A total of 1611 metabolites were identified, including 107 alkaloids, 276 amino acids and derivatives, 283 flavonoids, 86 lignans and coumarins, 181 lipids, 68 nucleotides and derivatives, 101 organic acids, 190 phenolic acids, 10 quinones, 4 steroids, 17 tannins, 111 terpenoids, and 177 other metabolites. Detailed information on all identified metabolites can be found in Supplementary Table S1.

### PCA and OPLS-DA for four *C. tuberculata* seeds

**Figure 2:**
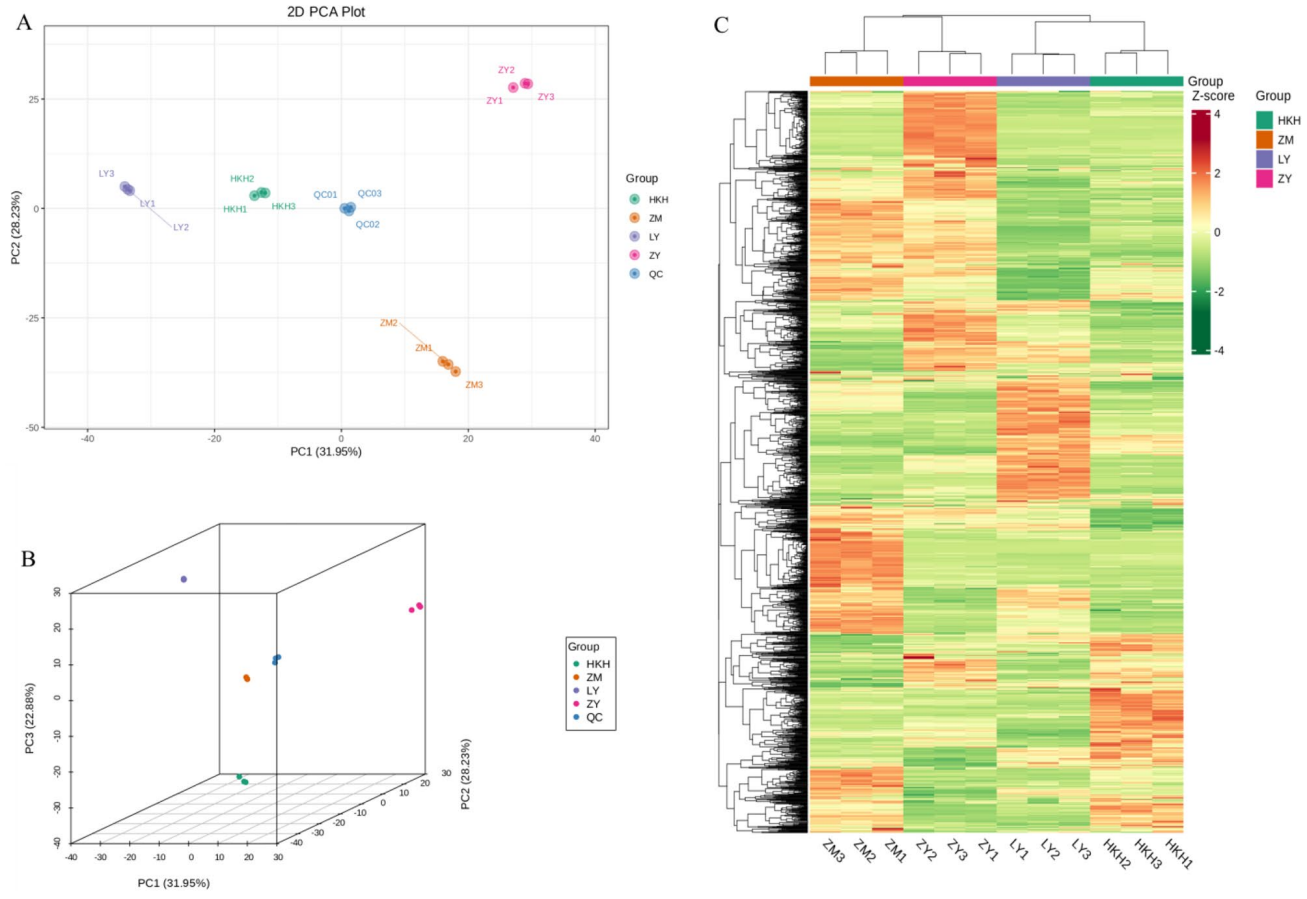
PCA and heat map analysis of metabolites in four *C. tuberculata* varieties [(**A**) PCA score plot. (**B**) Three-dimensional PCA plot. (**C**) Clustering heat map of all Metabolites].

To determine the overall metabolic differences between groups of samples of different *C. tuberculata* cultivars and the metabolite differences between variability of samples within groups, PCA and HCA analyses were first performed. In the present study, PC1 and PC2 accounted for 31.95% and 28.23% of the total variance, respectively. In addition, the four *C. tuberculata* varieties were clearly separated, and the three replicates of each variety were clustered together (Fig. 2A). All samples were classified into four groups based on metabolites, as follows: (1) HKH, (2) LY, (3) ZY, and (4) ZM. In the three-dimensional PCA plot, PC3 accounted for 22.88% of the total variation, further justifying the classification of the four seed samples into four groups (Fig. 2B). In addition, the PCA results showed relatively low variability among the biological replicates, reflecting the strong correlation among replicates. Consistent with the results of the PCA analysis, HCA also performed a cluster analysis of the metabolite accumulation patterns of the different samples, and the cluster lines on the left side of the figure represent the metabolite clustering (Fig. 2C). In addition, we used Orthogonal Partial Least Squares-Discriminant Analysis (OPLS-DA) to analyze the variable causes of the differences between these four groups. In this study, the Q2 values for all controls were above 0.9 (Supplementary Fig. S4), indicating that these models remained stable. The OPLS-DA score plot showed that the four cultivars were well separated pairwise, indicating significant differences in metabolic phenotypes among the four cultivars.

### Differential metabolite screening, functional annotation, and enrichment analysis among the four *C. tuberculata* varieties

#### Differential metabolite screening

**Figure 3:**
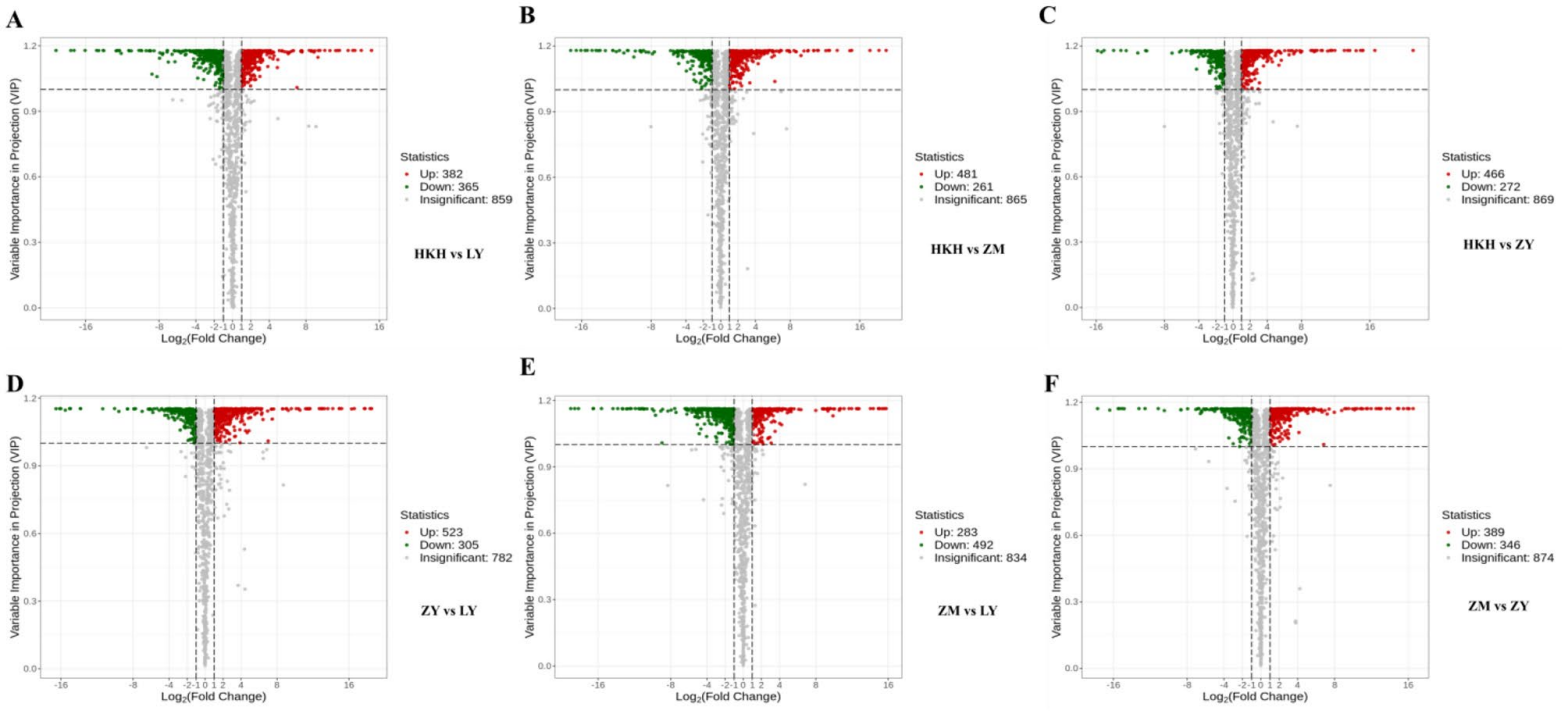
(**A**–**F**) Volcano plot between paired samples.

Based on OPLS-DA, metabolites with differences between species or tissues were first filtered out using the variables in the multivariate analysis model for VIP. It was also combined with univariate analysis to further search for different metabolites. The criteria for detecting differentially enriched metabolites were: Fold-change ≥ 2 and ≤ 0.5, and VIP ≥ 1. In this study, six pairwise comparisons of metabolite contents of seed samples were performed using Fold-change and VIP values. The results showed differences between species with values ranging from 45.68% to 51.43%; there were 747 significantly different metabolites between KHK and LY (382 upregulated, 365 downregulated), 742 between HKH and ZM (481 upregulated, 261 downregulated), 738 between HKH and ZY (466 upregulated, 272 downregulated), 828 between ZY and LY (523 upregulated, 305 downregulated), 775 between ZM and LY (283 upregulated, 492 downregulated), and 735 between Red and Black (389 upregulated, 346 downregulated) (Supplementary Table S2, Fig. 3).

The relative contents of all the differential metabolites in the control group were subjected to K-means clustering analysis to reveal the trends of metabolite contents in different subgroups. The results showed that a total of 1311 differential metabolites were identified based on standardized relative metabolite levels. These metabolites included 77 alkaloids, 239 amino acids and derivatives, 255 flavonoids, 82 lignans and coumarins, 133 lipids, 59 nucleotides and derivatives, 80 organic acids, 153 phenolic acids, 8 quinones, 4 steroids, 6 tannins, 96 terpenoids, and 119 other metabolites. The 1311 identified differential metabolites were classified into three subclasses by the K-mean method, and the content of metabolites in these subclasses differed (Supplementary Fig. S5).

#### Key significantly differential metabolites

To identify the major metabolites of different seeds, we plotted the relationship between the groups of different metabolites using four *C. tuberculata* seeds (KHK, LY, ZM, ZY) by constructing a Venn diagram. The results showed that a total of 63 overlapping significantly different metabolites were identified in six two-to-two comparisons (Supplementary Fig. S6A). There were 261 overlapping differential metabolites identified in the two-for-two comparison of HKH with ZM, LY, and ZY (Supplementary Fig. S6B), 298 overlapping metabolites identified in the two-for-two comparison of ZM with HKH, LY, and ZY (Supplementary Fig. S6C), and a total of 287 overlapping metabolites identified in the three two-for-two comparisons of ZY with HKH, LY, and ZM with LY (Supplementary Fig. S6D). The 63 overlapping metabolites examined included 3 alkaloids, 11 amino acids and derivatives, 10 flavonoids, 12 lignans and coumarins, 4 lipids, 2 organic acids, 7 phenolic acids, 10 terpenoids, and 4 other metabolites, with the highest proportion of lignans and coumarins (19%) (Supplementary Fig. S6E, Supplementary Table S3).

#### KEGG pathway annotation of the differential metabolites

**Figure 4:**
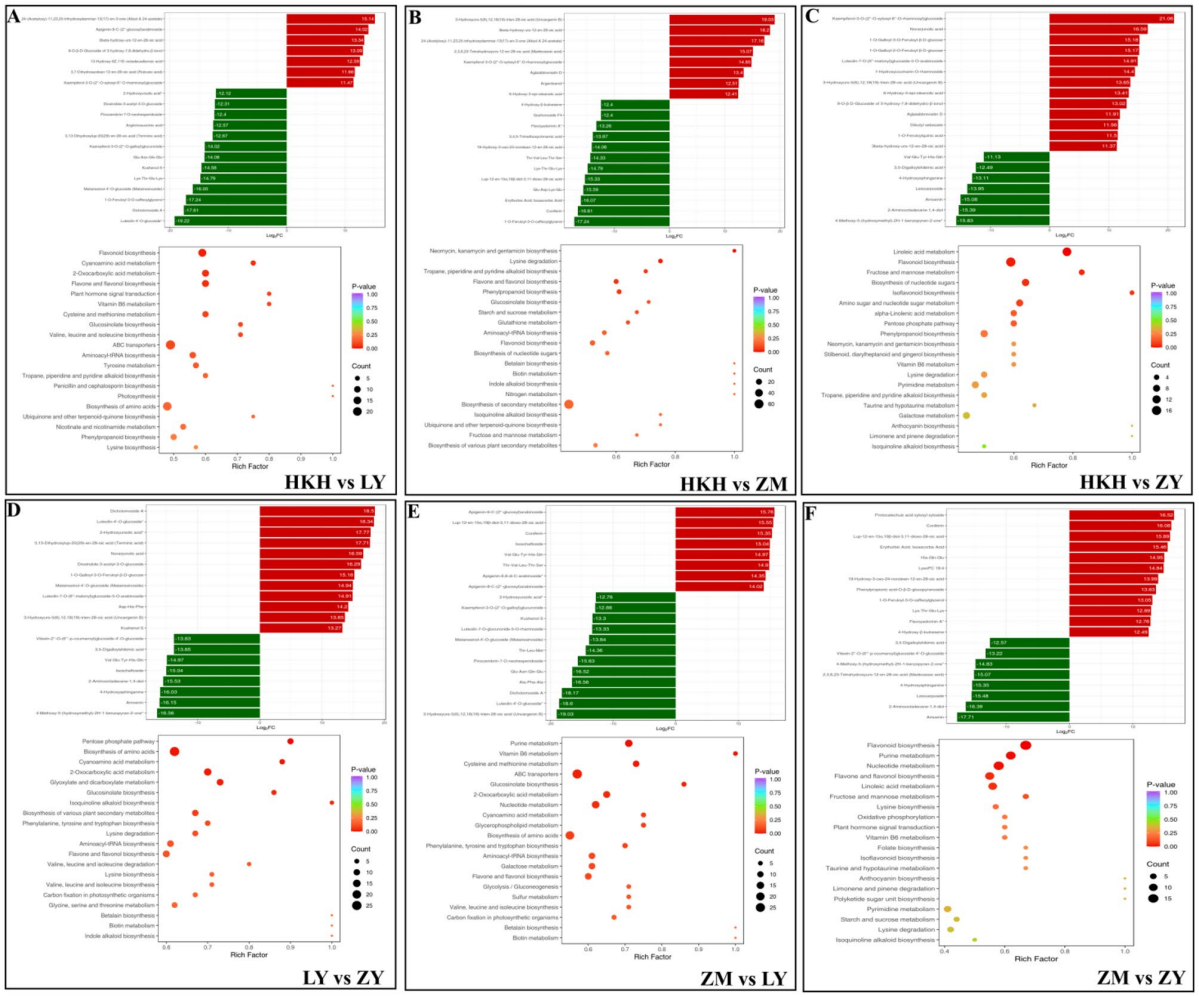
Bar graphs of differentially accumulated metabolites and KEGG enrichment bubbles for each subgroup. [(**A**) HKH vs LY; (**B**) HKH vs ZM; (**C**) HKH vs ZY; (**D**) LY vs ZY; (**E**) ZM vs LY; and (**F**) ZM vs ZY. The upper part of the graph shows the results of the top 20 metabolites in each group comparison; the red color represents the up-regulation of metabolites, and the green color represents the down-regulation of metabolite content. In the lower part of the graph, the color of the dots reflects the p-value size; the redder the color].

The annotation results for the differentially significant KEGG metabolites were classified into three categories according to the similarity of the pathways in KEGG: Metabolism, Genetic Information Processing, Environmental Information Processing (Supplementary Fig. S7). After the qualitative and quantitative analysis of the detected metabolites, the change in the frequency of the differences in the quantitative information of the metabolites that occurred in each group was compared in relation to the grouping of the specific samples. Based on the differential metabolite results, KEGG pathway enrichment analysis was performed, where the Rich factor is the ratio of the number of differential metabolites in the corresponding pathway to the total number of metabolites annotated for the pathway, and the higher the value, the higher the enrichment level. The top 20 metabolic pathways, ranked by the p value of the hypergeometric test, were selected for display, from the largest to the smallest. Their results showed (Fig. 4) that the differentially accumulated metabolites were enriched in different metabolic pathways between HKH vs. LY, HKH vs. ZM, HKH vs. ZY, LY vs. ZY, ZM vs. LY and ZM vs. ZY, with the most enriched pathways being 4, 2, 4, 7, 7, and 5 respectively (p-value < 0.05); furthermore, when comparing subgroups with different abundance of top metabolites, 7, 8, 13, 12, 8, 12 metabolites were upregulated and 13, 12, 7, 8, 12, 8 metabolites among top 20 metabolites were downregulated in each group comparison. Differential accumulation of metabolites in HKH vs. LY was mainly enriched in flavonoid biosynthesis, cyanoamino acid metabolism, 2-oxocarboxylic acid metabolism, and flavone and flavonol biosynthesis; differential accumulation of metabolites in HKH vs. ZM was mainly enriched in neomycin, kanamycin, and gentamicin biosynthesis and lysine degradation; the differential accumulation of metabolites in HKH vs. ZY was mainly enriched in linoleic acid metabolism, flavonoid biosynthesis, fructose and mannose metabolism, and nucleotide sugar biosynthesis; the differential enrichment of metabolites in LY compared with ZY was mainly enriched in penta-synthesis. ZY was mainly enriched in the pentose phosphate pathway, biosynthesis of amino acids, cyanoamino acid metabolism, 2-oxocarboxylic acid metabolism, glyoxylate and dicarboxylate metabolism, biosynthesis of glucosinolates, and biosynthesis of isoquinoline alkaloids; the differential enrichment of metabolites in ZM vs. LY was mainly enriched in purine metabolism, vitamin B6 metabolism, cysteine and methionine metabolism, ABC transporters, glucosinolate biosynthesis, 2-oxocarboxylic acid metabolism, and nucleotide metabolism; the differential enrichment of metabolites in ZM vs. ZY was mainly enriched in flavonoid biosynthesis, purine metabolism, nucleotide metabolism, flavone and flavonol biosynthesis, and linoleic acid metabolism.

### Analysis of TPC, TFC and antioxidant capacity

**Figure 5:**
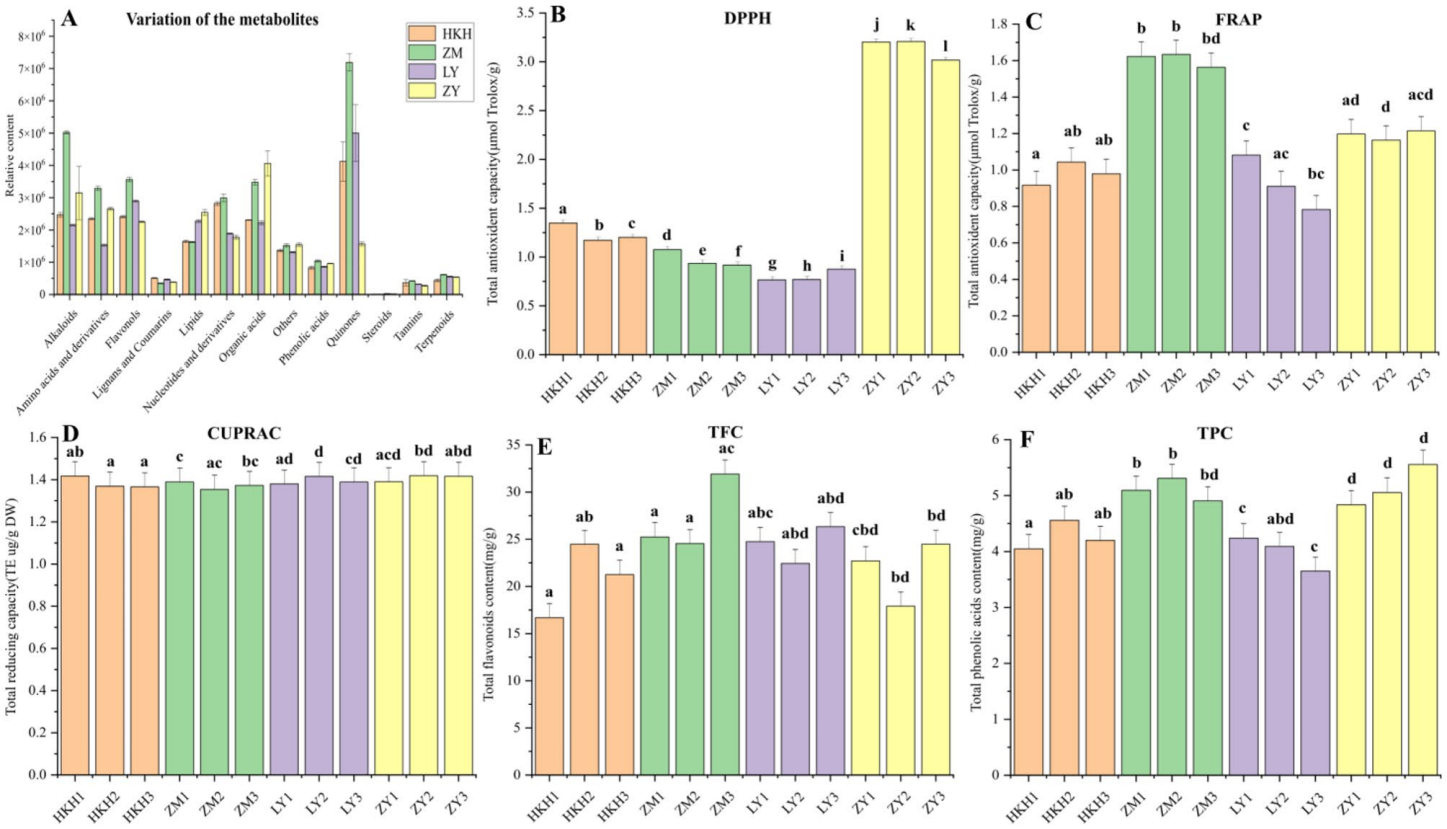
The relative content of each class of metabolites and antioxidant activities of the different seeds [Comparison of the relative content of each category of metabolites in KHK, ZM, LY, and ZY (**A**); antioxidant activities of the different colored seeds using DPPH assay (**B**), FRAP assay (**C**), CUPRAC assay (**D**), TFC assay (**E**), and TPC assay (**F**). Different letters above bars indicate statistically significant differences at *p* < 0.05].

The results of correlation analysis between TPC and TFC of DPPH, FRAP and CUPRAC of *C. tuberculata* seeds showed that TPC showed significant positive correlation (*p* = 0.518) and highly significant positive correlation (*p* = 0.790) with DPPH and FRAP, respectively; TFC showed significant negative correlation (*p* = − 0.526) with CUPRAC (Table S4). To investigate the antioxidant capacity of the different seeds of *C. tuberculata*, we evaluated the antioxidant activity of the seeds by comparing the content of different metabolites between the different seeds and using three methods: DPPH, CUPRAC, and FRAP. The results showed that the alkaloids, amino acids and derivatives, flavonols, lignans and coumarins, lipids, nucleotides and derivatives, organic acids, others, phenolic acids, quinones, steroids, tannins and terpenoids of the different seeds were different; Alkaloids, amino acids and derivatives, flavonols, nucleotides and derivatives, quinones, phenolic acids, tannins, and terpenoids were different in ZM derivatives; quinones, phenolic acids, tannins, and terpenoids were higher in ZM than in the other seeds; organic acids were highest in ZY; LY and ZY had higher error lines for alkaloids and Quinones (Fig. 5A). The antioxidant properties of ZM and LY were higher than those of HKH and ZY (Fig. 5B–D). The TPC and TFC content was highest in ZM (Fig. 5E,F).

### Identification of anti-disease active pharmaceutical ingredients and key active ingredients of traditional Chinese medicine for seven diseases of *C. tuberculata* seeds

**Figure 6:**
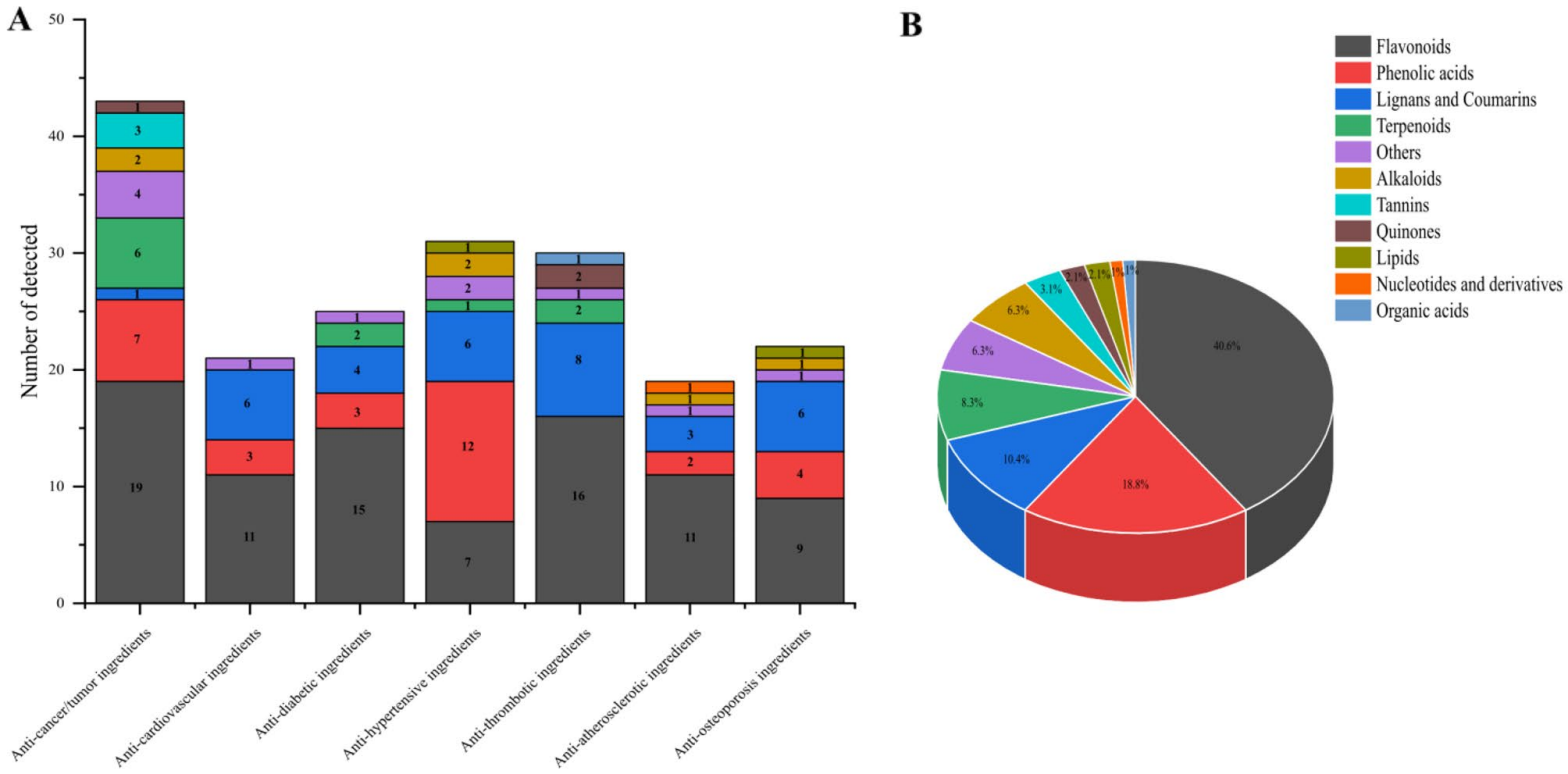
Identification of active components of *C. tuberculata* seed metabolites for disease resistance [(**A**) detection of disease-resistant metabolite species for various diseases; (**B**) classification of 96 disease-resistant metabolites A].

Cancer/tumors, diabetes, hypertension, cardiovascular disease, atherosclerosis, blood clots and osteoporosis diseases are currently the major threats to human health worldwide. To identify the active pharmaceutical components of *C. tuberculata* seeds against these seven major diseases, we used the metabolites identified in this study for comparison of disease resistance components through the TCMSP database. A total of 96 metabolites with disease-resistant properties were identified, including anti-cancer/tumor, anti-cardiovascular, anti-diabetic, anti-hypertensive, anti-thrombotic, anti-atherosclerotic, and anti-osteoporotic constituents (43, 21, 25, 31, 30, 19, and 22, respectively) (Fig. 6A, Supplementary Table S5). And the 96 metabolites were classified into 11 categories, including alkaloids, flavonoids, lipids, lignans and coumarins, nucleotides and derivatives, organic acids, others, phenolic acids, quinones, tannins, and terpenoids, numbered 6, 39, 2, 10, 1, 6, 18, 2, 3,and 4, respectively (Fig. 6B, Supplementary Table S5). In addition, we also analyzed the main active ingredients of 96 traditional Chinese medicine metabolites and counted the related disease numbers, related target numbers, oral bioavailability, and drug likenesses (Supplementary Table S6). Interestingly, we found that some metabolites may appear in multiple diseases, e.g., isorhamnetin-3-*O*-glucoside and quercetin in 7 diseases, vitexin and dehydrodiisoeugenol in 6 diseases, nobiletin and fraxetin in 5 diseases, resveratrol in 4 diseases, rutin in 3 diseases, embelin and ursolic acid in 2 diseases (Supplementary Table S5).

## Discussion

*Camellia tuberculata* belongs to the genus *Camellia*. Due to its peculiar morphological features, it has increasingly attracted the attention of scientists. However, the development and utilization of this group of plants is limited by the lack of clarity regarding the composition of its inherent metabolites. To date, studies on *C. tuberculata* have mainly examined the leaves for specific metabolites, such as catechin-like compounds, theanine, and caffeine in the leaves, while little has been reported on the seeds^36^. Metabolomics is the large-scale study of small molecules in cells, tissues, or organisms to quantify the multivariate dynamic responses of living organisms to external stimuli, pathophysiological changes, and their own genetic mutations at the level of their metabolites^37^. Therefore, in the present study, the four seeds of *C. tuberculata* were analyzed by UPLC/ESI-Q TRAP-MS/MS to establish a comprehensive metabolic profile. A total of 1611 metabolites were identified, most of which are phytochemicals. So far, most alkaloids, amino acids and derivatives, flavonoids, lipids, nucleotides and derivatives, phenolic acids, steroids, tannins, and terpenoids have been detected in *C. tuberculata*^38,39^. The number of metabolites identified was much higher than the number of metabolites from cannabis, proso millet, sesame, and buckwheat seeds^8,40–42^. A large number of bioactive components have also been identified, which will play an important role in exploring the medicinal and nutritional value of the seeds.

In this study, the values of the variables importance in the projection (VIP) of the multivariate analysis model were used for the preliminary screening of differential metabolites between two pairs of four *C. tuberculata*. A total of 1311 differential metabolites were screened, of which 382, 481, 466, 523, 283, 389 were upregulated and 365, 261, 272, 305, 492, 346 were downregulated in HKH vs. LY, HKH vs. ZM, HKH vs. ZY, LY vs. ZY, ZM vs. LY, and ZM vs. ZY, respectively. The 1311 differential metabolites studied were also divided into three subclasses by the K-means method, and the content of metabolites in these subclasses differed. The major metabolites of the different seeds were identified by constructing a kind of Wayne diagram. In six two-by-two comparisons, a total of 63 overlapping, significantly different metabolites were found, which may be the major metabolites differentially regulated in *C. tuberculata* seeds during development. In the six two-by-two comparison groups, the differentially accumulated metabolites exhibited different metabolic pathways. In particular, 2-oxocarboxylic acid metabolism (p-value < 0.05) was observed in all comparisons of LY with HKH, ZY, and ZM. 2-Oxocarboxylic acid plays an important role in energy production, biosynthesis, carboxylic acid cycle, and thermal adjustment regulation, and is essential for normal plant growth and development^43,44^. Therefore, this will provide new insights to study the growth, development, and systematic classification of *C. tuberculata* plants.

Plant flavonoids are a class of natural plant compounds with antioxidant, anti-inflammatory, anti-allergic, lipid-lowering, anti-cancer, neuroprotective, anti-aging, and other beneficial functions for human health^45–47^. At the same time, phenolic acids contained in plants can not only scavenge free radicals and protect cells from oxidative damage, but also protect the cardiovascular and cerebrovascular systems, inhibit tumor growth, improve immune function, etc^48,49^. The phenylalanine pathway is the main pathway for the synthesis of phenolic acids in most plants; however, gallic acid is synthesized via the mangiferous acid pathway. Gallic acid is present in high concentrations in most plants of the genus *Camellia*. Previous studies have shown that different concentrations of methanolic extracts correlate differently with DPPH^50^. In the present study, TPC extracted with 60% methanol showed significant correlation with DPPH, which is consistent with most studies^51^. TPC showed highly significant correlation with FRAP, which is in agreement with the results of Tang et al. on lily antioxidants^52^. Therefore, the results of the antioxidant capacity test may be slightly different for the same sample using different extraction methods^53^. The results of DPPH, FRAP, and CUPRAC analyses showed that ZM had the highest DPPH content and ZY had the highest FRAP content, and that ZM and ZY had the highest phenolic acid content among the four *C. tuberculata* seeds. In conclusion, our results show that all tested seed extracts have strong antioxidant activity. Therefore, we hypothesize that the phenolic acid compound content may be the main factor for the antioxidant activity of the four *C. tuberculata* seeds and that the high antioxidant activity of ZM and ZY can be used as a future study for disease prevention, health care, etc.

The seeds of the genus *Camellia* have high oil content and are important as industrial and edible oils. In addition, the genus *Camellia* has potential medicinal value because it contains mainly saponins, tannins, and flavonoids. The main pharmacological effects are anticancer, antibacterial, antiosteoporosis, cardiovascular and cerebrovascular diseases, etc.^54,55^. Therefore, we analyzed seven disease-resistant pharmaceutical agents based on several identified metabolites, and 96 disease-resistant pharmaceutical agent metabolites were identified. Forty-eight metabolites with an oral bioavailability (OB) ≥ 5% and drug-likeness (DL) ≥ 0.14 were investigated, and these were considered to be important traditional Chinese medicine active ingredients from *C. tuberculata* seeds. Among the 96 metabolites identified, flavonoids and phenolic acids were the most diverse, which may be the main reason for the high disease resistance of *C. tuberculata* seeds. Therefore, we believe that *C. tuberculata* seeds have important potential medicinal value and further confirm the authenticity of the pharmacological effects of the genus *Camellia*, such as fighting cancer, bacteria, osteoporosis, and cardiovascular diseases.

## Conclusions

This study is the first to investigate the metabolites of *C. tuberculata* seeds based on UPLC/ESI-Q TRAP-MS/MS with comprehensive targeted metabolomics. A total of 1,611 metabolites were identified, including mainly alkaloids, amino acids and derivatives, flavonoids, lignans and coumarins, lipids, nucleotides and derivatives, organic acids, phenolic acids, quinones, steroids, tannins, and terpenoids. The 1311 different metabolites identified were classified into three categories. sixty-three overlapping significantly different metabolites were identified in six two-to-two comparisons. KEGG pathway enrichment for different metabolites showed that the different accumulated metabolites had different metabolic pathways in HKH vs. LY, HKH vs. ZM, HKH vs. ZY, LY vs. ZY, ZM vs. LY and ZM vs. ZY, with 2-oxocarboxylic acid metabolism observed in the comparison of LY with HKH, ZY and ZM. The results of the analysis of total flavonoids, total phenolic acids, and antioxidant capacity of the four *C. tuberculata* species showed that the content of phenolic acid compounds was the most important factor of antioxidant activity of the four *C. tuberculata* seeds. In addition, the 96 identified antipathogenic pharmaceutical agents play an important role in combating cancer, bacteria, osteoporosis, and cardiovascular and cerebrovascular diseases. In conclusion, this study not only reveals the major metabolites and antioxidant and disease inhibitory pharmaceutical components in *C. tuberculata* seeds, but also provides important clues to the development and utilization of its medicinal value, woody oil crops, and ornamental species for gardens.

### Supplementary Information

Supplementary Figures.

Supplementary Tables.

## Data Availability

The authors confirm that the data supporting the findings of this study are available within the article.
