# Supplementary material for: Extensive targeted metabolomics analysis reveals the identification of major metabolites, antioxidants, and disease-resistant active pharmaceutical components in Camellia tuberculata (Camellia L.) seeds
Source: Sci Rep. 2024 Apr 15;14:8709. doi: 10.1038/s41598-024-58725-0 (PMC11018803; doi:10.1038/s41598-024-58725-0)
Supplement: Supplementary file 1 — Supplementary Figures. [file 41598_2024_58725_MOESM1_ESM.docx]

**Supplementary Figure**


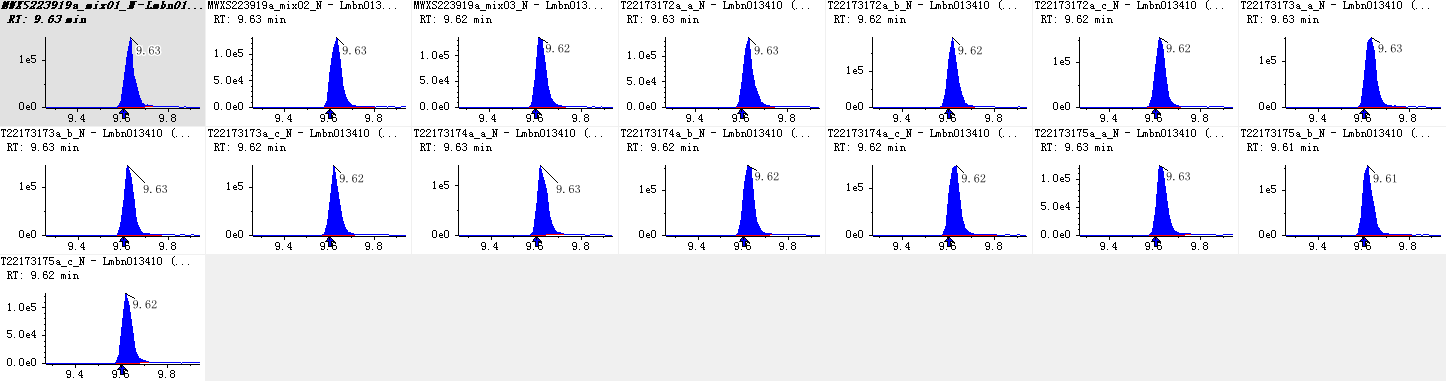

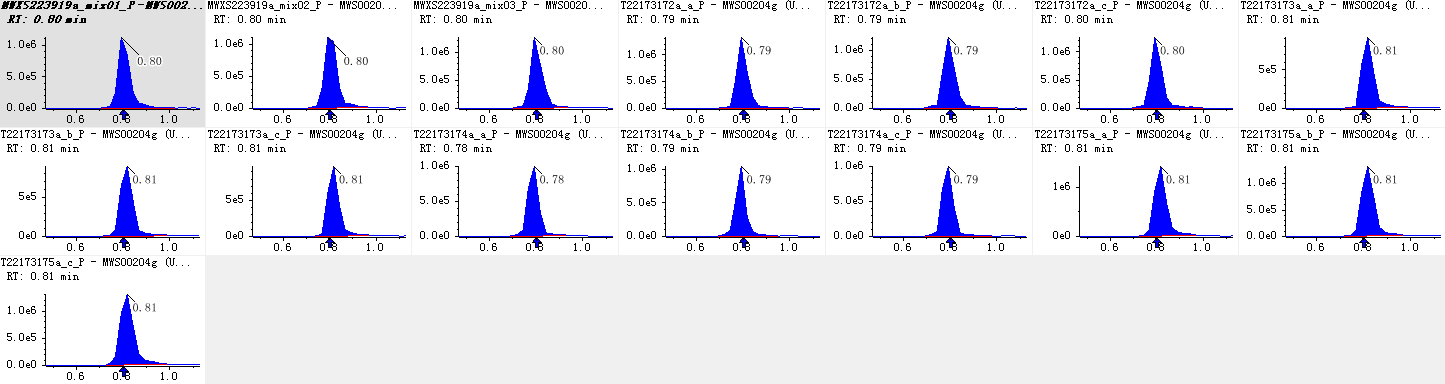


**Figure S1.** Integration correction plots for quantitative analysis of randomly selected metabolites in different samples. (The horizontal coordinate is the retention time (min) for metabolite detection, and the vertical coordinate is the ion flow intensity (cps) for ion detection of a metabolite.)


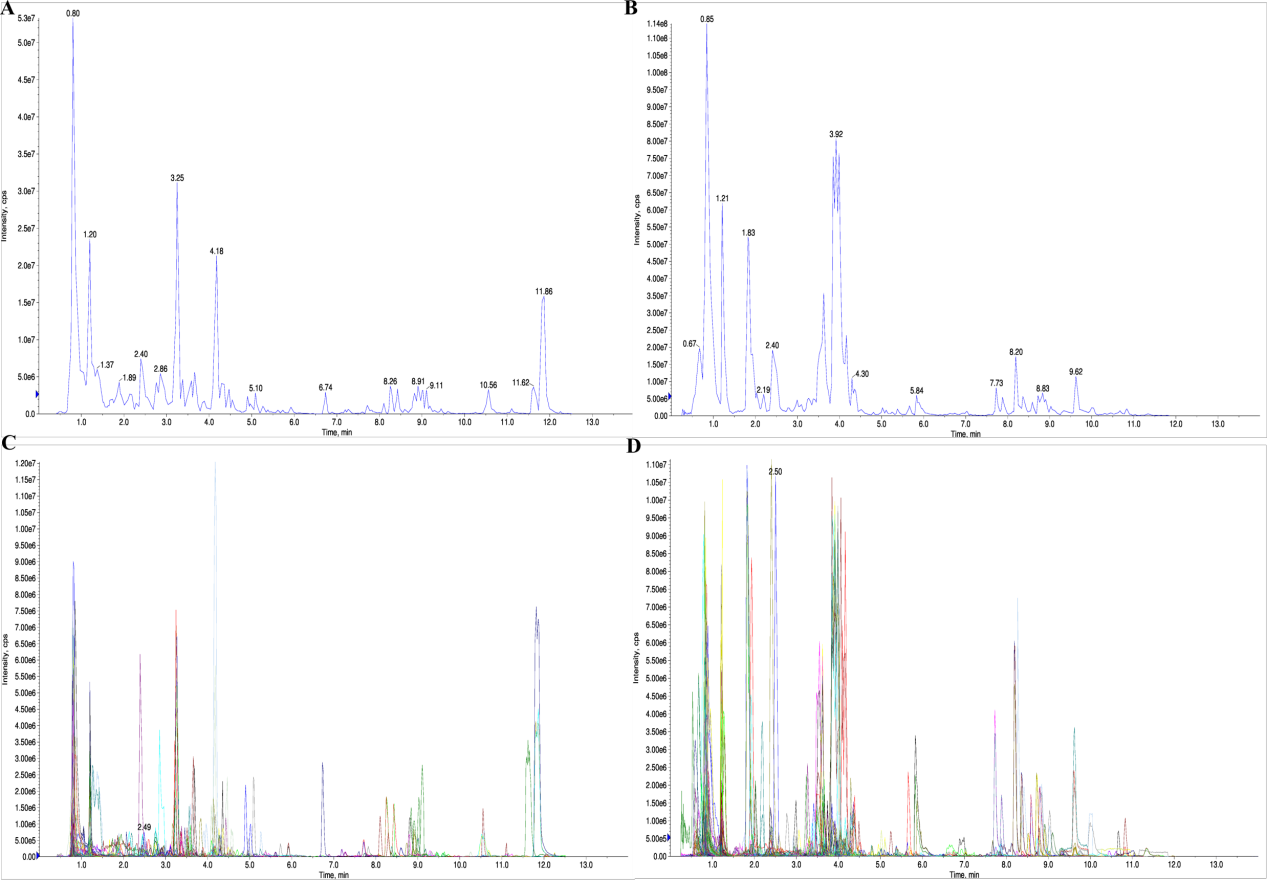


**Figure S2.** Total ion current of one quality control sample by mass spectrometry detection (**A**, **B**) and multi-peak detection plot of metabolites in the multiple reaction monitoring mode (**C**, **D**). **A** and **C** were acquired in Negative ionization mode. **B** and **D** were acquired in positive ionization mode. (TIC indicates the plot made by summing the intensities of all ions in the mass spectrometry plot at each time point against time; MRM indicates the materials that can be detected in the sample; and each differently colored peak represents one metabolite detected.)


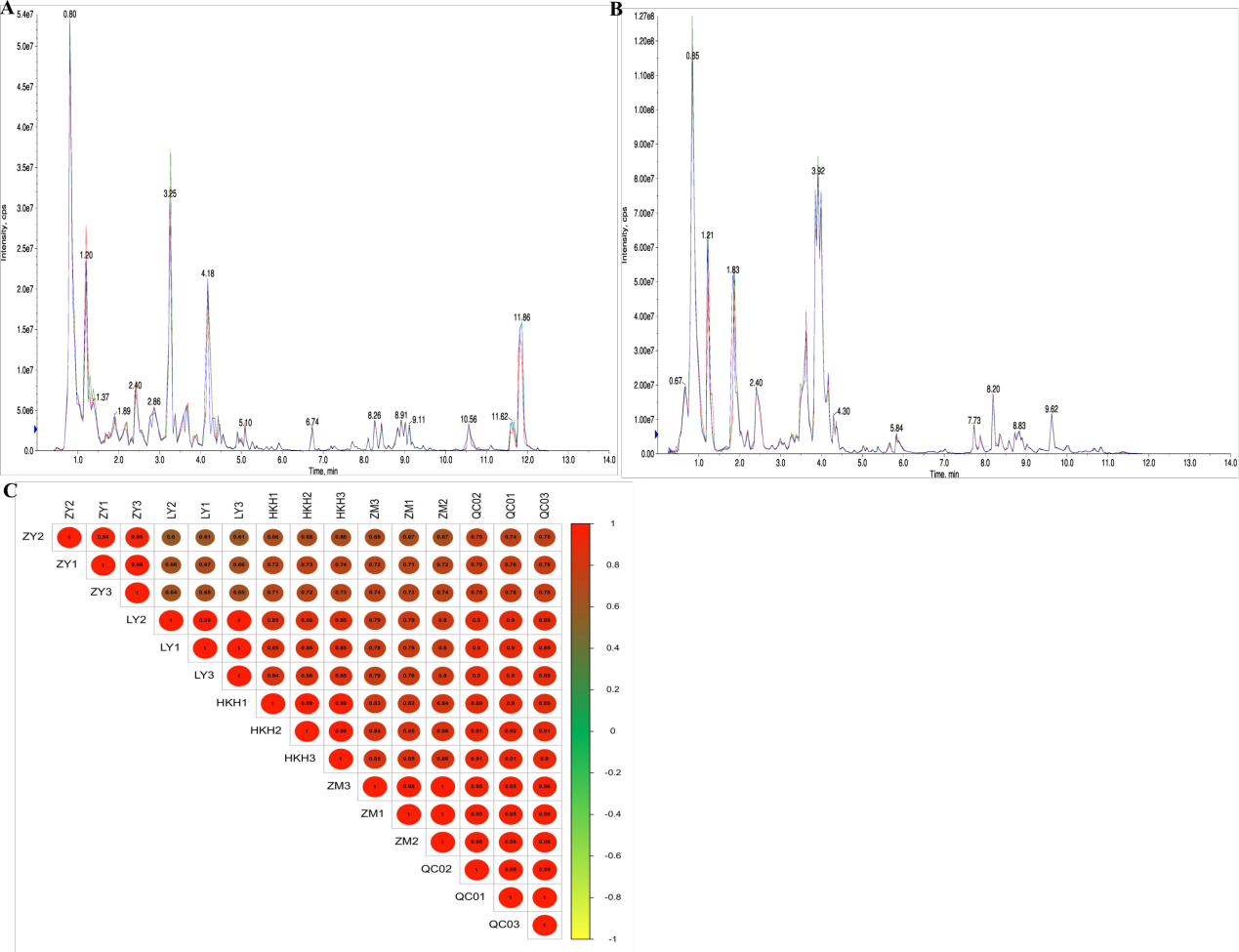


**Figure S3.** Total ions current overlaps of the three quality control samples by mass spectrometry detection. (A, TIC overlay plot in negative ionization mode. B, TIC overlay plot in positive ionization mode. C, correlation analysis of all the samples. three replicate samples (1, 2, 3). QC: quality control samples.)


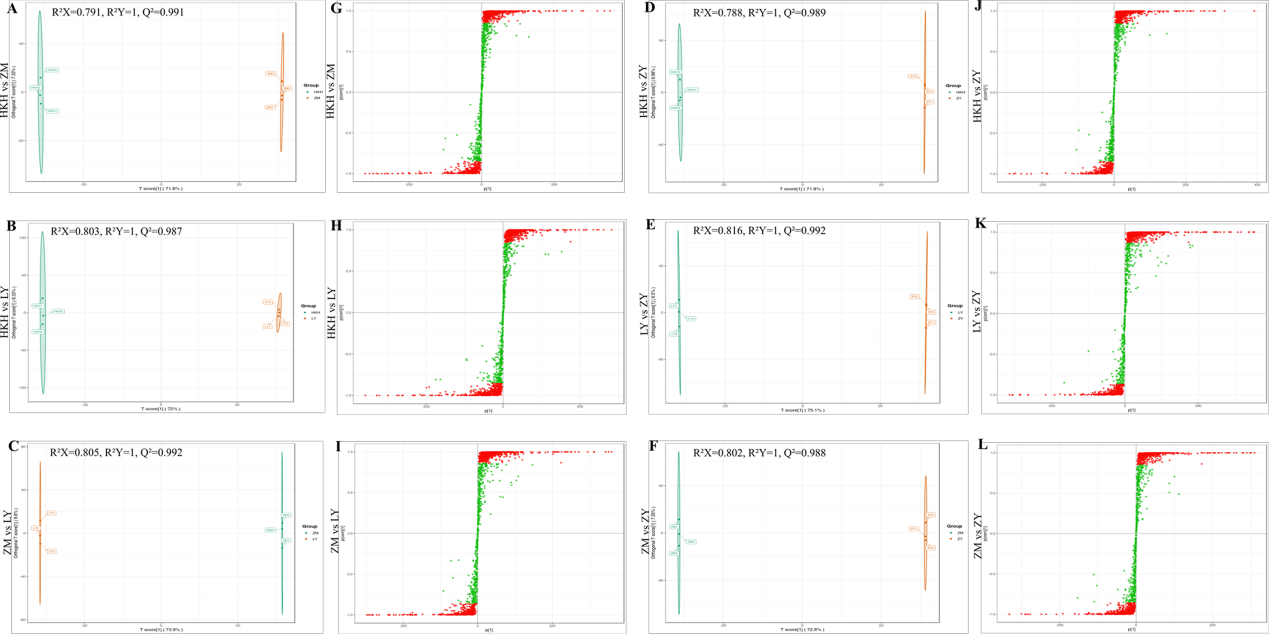


**Figure S4.** The OPLS-DA score plots of four *Camellia tuberculata* varieties. (A-F, OPLS-DA model plots for the comparison groups HKH vs. ZM, HKH vs. LY, ZM vs. LY, HKH vs. ZY, LY vs. ZY, and ZM vs. ZY, respectively.)


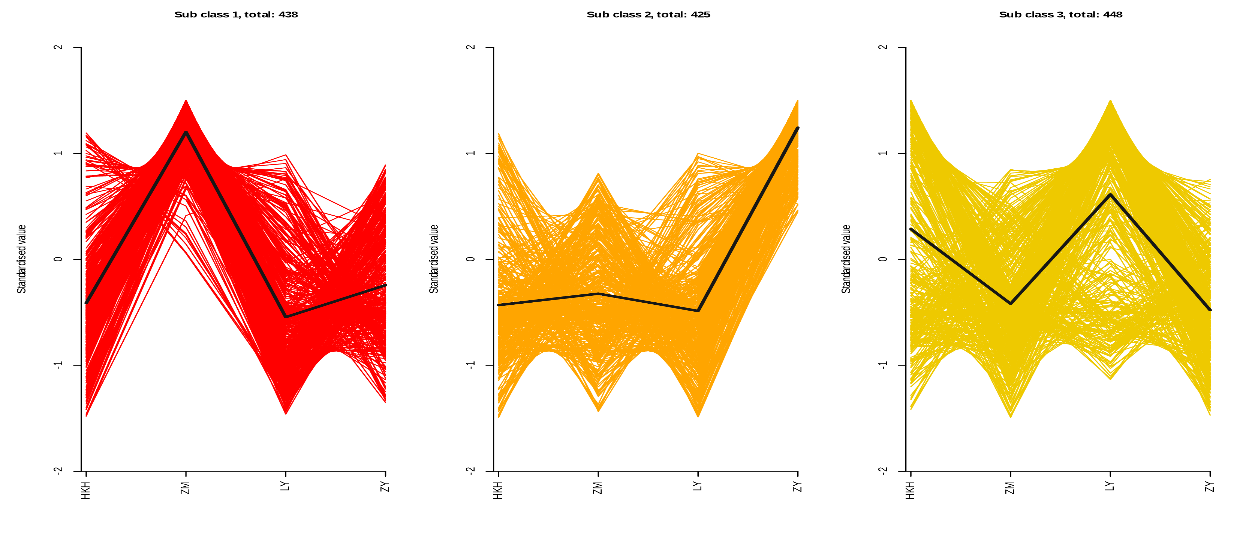
**Figure S5.** K-Means plots of four different metabolites of *C. tuberculata*.

_
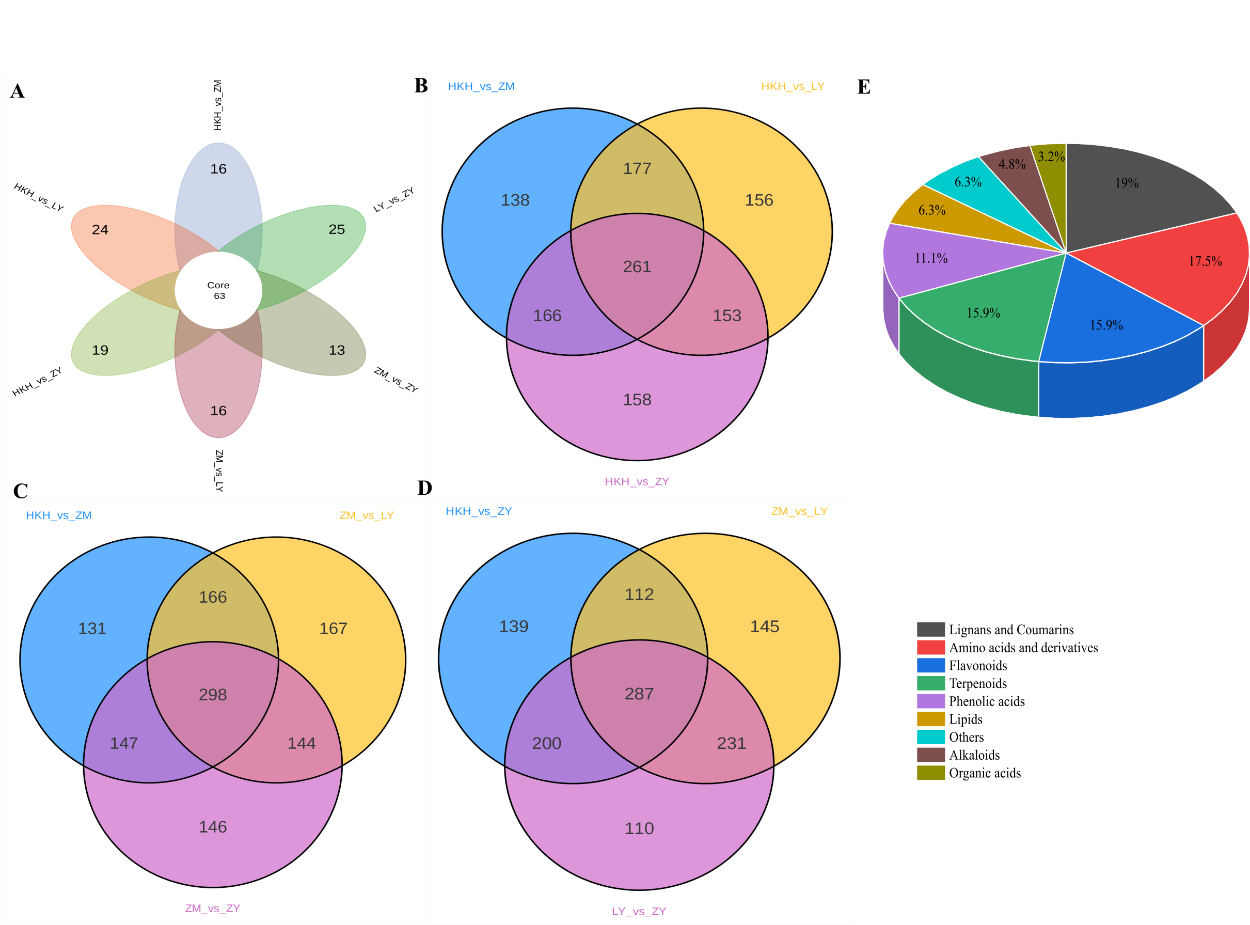
_

**Figure S6.** Venn diagram of differential metabolites in each group. ((A) HKH vs ZM, HKH vs LY, HKH vs ZY, ZM vs LY, ZM vs ZY, and LY vs ZY; (B) HKH vs ZM, HKH vs LY, and HKH vs ZY; (C) ZM vs HKH, ZM vs ZY, and ZM vs LY; (D) HKH vs ZY, LY vs ZY, and ZM vs LY; and (E) Classification of the 63 key metabolites. Each circle in the graph represents a comparison group, and the numbers in the overlapping part of the circles represent the number of differential metabolites shared between the comparison groups, while the numbers without the overlapping part represent the number of differential metabolites specific to the comparison groups.)


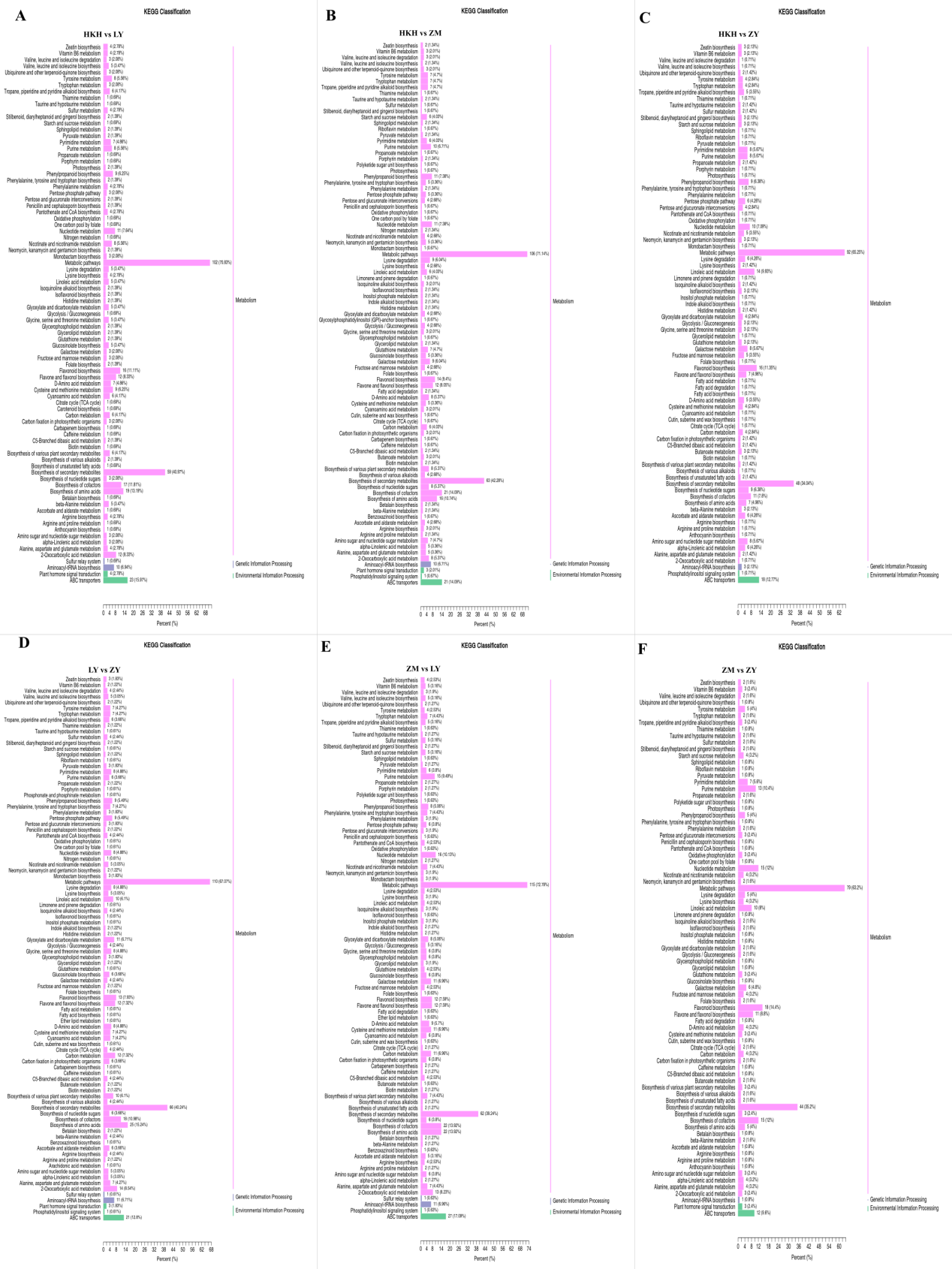


**Figure S7.** Differential metabolite KEGG classification chart. (The vertical coordinate is the name of the KEGG metabolic pathway, and the horizontal coordinate is the number of differentials annotated to that pathway and their number as a proportion of the total number of differential metabolites annotated.)
